# 16 Months Follow Up of Patients’ Behavior and Mild COVID-19 Patterns in a Large Cohort of Cancer Patients During the Pandemic

**DOI:** 10.3389/fonc.2022.901426

**Published:** 2022-06-07

**Authors:** Nawale Hajjaji, Kathleen Lepoutre, Sarra Lakhdar, Stéphanie Bécourt, Charlotte Bellier, Emilie Kaczmarek, Antonin Broyelle, Sandrine Giscard, Eric Lartigau

**Affiliations:** ^1^Medical Oncology department, Oscar Lambret Cancer Center, Lille, France; ^2^Inserm, U1192, Laboratoire Protéomique, Réponse Inflammatoire et Spectrométrie de Masse (PRISM), Univ. Lille, Lille, France; ^3^Quality department, Oscar Lambret Cancer Center, Lille, France; ^4^Radiotherapy department, Oscar Lambret Cancer Center, Lille, France

**Keywords:** cancer patients, mild COVID-19 infection, long COVID, patients’ behavior, shielding measures, anti-COVID-19 vaccination

## Abstract

**Background:** Acute severe forms of COVID-19 infection are more likely in cancer patients and growing attention has been given to the persistent symptoms of the disease after severe COVID-19. However, mild illness is the dominant clinical presentation of COVID-19 infection. To investigate patients’ behavior and the short- and longer-term pattern of the disease in cancer patients with mild COVID infection, a longitudinal online survey was conducted for 16 months during the pandemic in a large cohort of cancer patients from a French COVID-19 hot spot. An online questionnaire was administered at three time points between the first wave of the pandemic in France and the fourth wave. The questionnaire was completed by 1415 to 2224 patients, which queried patients’ demographics, their behavior, and COVID infection patterns. Seventy percent of the patients were female, and 40% had a comorbid condition. More than one-third of the participants had breast cancer, and half were survivors. The rate of infection was 30% during wave 1 and 10% in wave 4; most patients had a mild COVID-19 infection. Twenty-five percent of infected patients during wave 4 did not seek medical advice. At wave 4, 87% of the patients received at least one dose of vaccine. Systematic compliance to shielding measures decreased over time. The short-term pattern of mild COVID changed between wave 1 and wave 4. Twenty-two percent of infected patients experienced persistent signs for more than 6 months with a negative impact on sleep, social behavior, and increased consumption of stress-relieving drugs. Our results showed a high prevalence of long-lasting symptoms in cancer patients with mild COVID-19 infection and inadequate behavior toward the disease and prevention measures among patients.

## Introduction

As the COVID-19 global pandemic marked 2 years, growing evidence showed the vulnerability of cancer patients. On top of its impact on access and quality of care, acute severe and fatal forms of the infection are more likely in cancer patients (1–3). The continuous adherence of cancer patients to risk protection measures is therefore paramount. However, their motivation is being put to the test by this unforeseeable infection. Growing attention has been given to persistent symptoms after COVID-19 infection (4, 5). Persistent signs are prevalent in up to 50% of patients with severe COVID in the general population (6, 7). However, mild-to-moderate disease constitutes the predominant symptomatic presentation of COVID-19 infection (8). Little is known about the short- and longer-term pattern of the disease in cancer patients with a mild COVID-19 presentation. And there is little information about how cancer patients reacted during the pandemic. To address this limited data, a longitudinal online survey was conducted to follow up a large cohort of cancer patients from a French COVID-19 hot spot, the Hauts-de-France region, for 16 months. The study investigated details regarding clinical symptoms of COVID-19 infection, patients’ behavior toward the infection, and the application of risk prevention measures during the pandemic. Our results showed up to 30% of cancer patients infected, mostly with mild COVID, and a high prevalence of long-lasting symptoms. Behavior toward the disease and prevention measures was inadequate among patients.

## Methods

### Study Design and Participants

**Figure 1:**
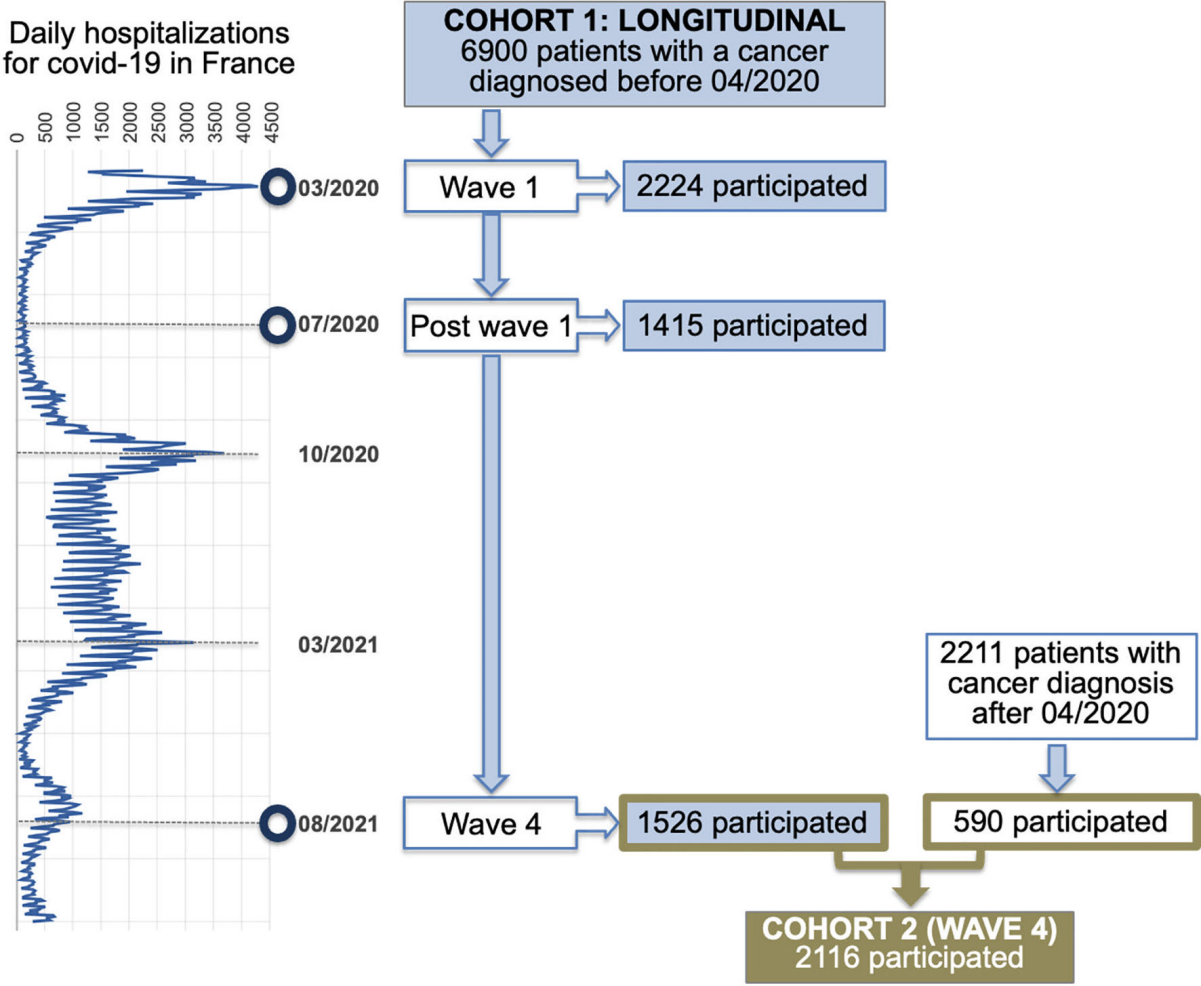
Study flow diagram. Cohort 1 is the longitudinal part of the study where online questionnaires were administered at three time points during the pandemic in France: the first wave, post wave 1, and wave 4. Cohort 2 gathered cancer patients from cohort 1 who completed the questionnaire at wave 4 and patients with newly diagnosed cancer during the pandemic (between May 2020 and July 2021). The left vignette shows the COVID-19 waves in France based on the curve of daily hospitalizations for COVID-19 nationwide.

This study was designed as an online survey to facilitate the large participation of cancer patients treated at Oscar Lambret Cancer Center (Lille, France) during the pandemic. This hospital is the comprehensive cancer center of the French region Hauts-de-France, which is a hotspot of COVID-19 infection in France. Two cohorts were conducted as shown in the flow diagram (**Figure 1**). Cohort 1 is a longitudinal study to investigate and follow up on details of clinical signs of covid-19 infection, the application of risk prevention measures, and the impact of the pandemic on patients’ feelings and behaviors. In cohort 1, online questionnaires were administered at three time points during the pandemic: the survey was launched in April 2020 during the first wave of COVID-19 in France, in July 2020 post wave 1, and in August 2021 during wave 4 of the pandemic in France. Cohort 2 was conducted to investigate COVID-19 symptoms lasting more than 6 months and patients’ behavior toward vaccination. Cohort 2 gathered cancer patients from cohort 1 who completed the questionnaire during wave 4 and patients with newly diagnosed cancer during the pandemic (between May 2020 and July 2021). At each time point, respondents were asked by email to fill in the survey, within a window of time of 2 weeks. All adult patients at Oscar Lambret center with an email address were invited to participate. Information about the objectives, the voluntary basis, anonymity, and data protection was provided online to the invited participants before the start of the questionnaires. Data were collected using the SurveyMonkey platform. Survey responses contained no personally identifiable information, and email addresses collected for the survey distribution were encrypted as anonymized participant IDs. The survey was reviewed by the local data protection officer and approved by our local institutional review board on April 16, 2020. The survey consisted of 20 questions and required a median time of 10 min to complete in order to improve participation and account for cancer, treatment, or COVID-19 symptoms that may limit sustained focus and attention. The survey was created and administered in French. In cohort 1, 6900 patients were invited to complete a questionnaire in April 2020, July 2020, and August 2021 with 2224, 1415, and 1526 respondents, respectively, as shown in the study flow diagram. Cohort 2 launched in August 2021 with 2116 patients participating (1526 from cohort 1 and 590 patients with newly diagnosed cancer) (**Figure 1**).

### Patients’ Demographics and Cancer Types

Patients’ age range, gender, BMI range, smoking habits, and comorbidities that have been associated with severe COVID-19 infection were collected, specifically chronic respiratory disease, hypertension, cardiac problems, asthma, diabetes, chronic renal failure, and cirrhosis. Information regarding cancer type and current care was queried.

### Epidemiology of COVID-19 Infection

We used the list of symptoms linked to COVID-19 infection communicated in March 2020 to health professionals by the French regulatory authorities. The list included fever, flu-like symptoms, skin rash, respiratory, digestive, and neurologic symptoms. The duration of the symptoms was explored with a focus on signs lasting more than 6 months in cohort 2, which included long COVID symptoms (5): fatigue, loss of taste or smell, muscle or joint pain, dizziness, respiratory symptoms, muscle weakness, sleeping troubles, headaches, cough, palpitations, loss of appetite, rhinitis or pharyngitis, hair loss, chest pain, digestive signs, ocular signs, fever or chills, skin rash, diagnosis of cardiovascular problems, diagnosis of thrombosis, diagnosis of diabetes, diagnosis of kidney dysfunction.

### COVID-19 Infection Management and Risk Prevention

We assessed whether infected patients sought medical advice during the pandemic. One item queried the type of medical visit: emergency room, general practitioner, or cancer center, and its format: physical visit or by web or phone. One question assessed whether patients had been tested for COVID, either using a PCR test or a serology. Since the only test available during the first wave was PCR, this was the only option in the initial questionnaire. A serologic test was added to the follow-up questionnaires. Using a Likert scale, one item assessed how cancer patients applied shielding measures defined as physical distance, wearing a mask, washing hands, coughing in their elbow, and staying at home.

### Impact of the Pandemic on Patients’ Behaviors

One item of the questionnaires assessed the impact of the pandemic on patients’ behaviors by querying specifically whether they were irritable, needed to self-isolate, had sleeping troubles, consumed more alcohol or tobacco, and consumed more stress-relieving drugs. Whether they felt security in their everyday life was also assessed by one item using a Likert scale. The answers “a little” and “not at all” were coded as insecurity. One item queried their need for psychological support and one item whether they were in financial difficulty (yes/no questions).

### Epidemiology of SARS-Cov2 Infection in the French Region Hauts-de-France

Information about the French COVID-19 national and regional data regarding shielding measures and vaccination were publicly available from the French government website GEODES (https://geodes.santepubliquefrance.fr).

### Statistical Analyses

We used descriptive statistics to analyze the baseline demographic information of the participants. A Chi2 test was used to compare categorical variables. Sankey diagrams were built with SankeyMatic (https://sankeymatic.com). P<0.05 was considered significant.

## Results

### Cohort Characteristics

Patients’ demographics, cancer type, and recent care are presented in **Table 1**. In cohort 1, 18% to 25% of the patients were younger than 50 years, 71% to 77% were female, 20% had a BMI over 30, 8% to 10% were current smokers, 17% to 24% were former smokers, 23% to 26% had hypertension, 8% to 9% had diabetes and cardiovascular disease, 3% to 5% had asthma, and 4% had a chronic respiratory disorder. The cancer-related questions revealed that 37% to 52% of the patients had breast cancer, 11% to 14% had gynecological cancer, and the other cancers (lung, head and neck, digestive, urologic, or sarcoma) constituted each less than 10% of the cohort; 47% to 58% were in follow-up; 8% to 13% received chemotherapy; 5% to 14% had radiation therapy; 7% to 12% had surgery; 12% to 14% received endocrine therapy; and less than 5% were treated with targeted therapy or immunotherapy. In cohort 2, the distribution of age, gender, BMI, smoking status, comorbidities, cancer type, and recent care were similar to cohort 1.

**Table 1:** Patients’ characteristics and COVID-19 infection in cohorts 1 and 2.

	Cohort 1					Cohort 2				
	Wave 1		Post wave		Wave 4		Newly diagnosed		Total cohort 2	
	n	%	n	%	n	%	n	%	n	%
Participants	2224		1415		1526		590		2116	
**Age (years)**										
<50	546	25	362	26	268	17	148	25	416	20
50-59	522	23	322	23	344	23	146	25	490	23
60-69	613	28	387	27	437	29	170	29	607	29
70+	494	22	288	20	447	29	113	19	560	26
Missing	49	2	56	4	30	2	13	2	43	2
**Gender**										
Female	1582	71	1009	71	1168	77	408	69	1576	75
Male	622	28	400	28	340	22	177	30	517	24
Missing	20	1	6	<1	18	1	5	1	23	1
**BMI (kg/m2)**										
<30	1715	77	1058	75	1169	76	434	73	1603	76
30+	464	21	302	21	299	20	128	22	427	20
Missing	45	2	55	4	58	4	28	5	86	4
**Cigarette smoking**										
Never-smoker	1473	66	907	64	1130	74	363	62	1493	71
Current smoker	204	9	148	11	126	8	60	11	186	9
Former smoker	531	24	344	24	256	17	161	27	417	20
Missing	16	1	16	1	14	1				
**Comorbidities**										
Hypertension	505	23	336	24	393	26	122	21	515	24
Diabetes	175	8	124	9	127	8	53	9	180	9
Cardiovascular diseases	157	7	109	8	143	9	45	8	188	9
Asthma	92	4	70	5	48	3	29	5	77	4
Chronic respiratory disorder	80	4	53	4	65	4	24	4	89	4
Chronic kidney disease	43	2	33	2	45	3	12	2	57	3
Cirrhosis	13	1	8	1	3	0	2	0	5	0
**Cancer type**										
Breast	971	44	_		800	52	217	37	1017	48
Gynecologic	237	11	_		172	11	80	14	252	12
Digestive	115	5	_		84	6	45	8	129	6
Head & neck	135	6	_		90	6	31	5	121	6
Lung	106	5	_		37	2	33	6	70	3
Urologic	155	7	_		105	7	38	6	143	7
Sarcoma	116	5	_		85	6	41	7	126	6
Missing	389	17	_		153	10	105	17	258	12
**Recent care**										
Follow up	1048	47	664	47	881	58	186	32	1067	50
Chemotherapy	295	13	147	10	126	8	163	28	289	14
Radiation therapy	315	14	147	10	78	5	123	21	201	9
Surgery	270	12	153	11	101	7	132	22	233	11
Endocrine therapy	312	14	174	12	183	12	78	13	261	12
Targeted therapy	82	4	44	3	44	3	44	7	88	4
Immunotherapy	51	2	34	2	22	1	27	5	49	2
Missing	175	8	197	14	215	14	105	18	320	15
**Clinical signs of infection**										
Yes	658	30	299	21	142	9	58	10	200	10
No	1479	66	1026	73	1367	90	526	89	1893	89
Missing	87	4	90	6	17	1	6	1	23	1
**Tested among patients with symptoms**	31	5	110	37	124	87	51	88	175	88
Positive test (PCR/Serology)	5	1	6	2	119	84	49	84	168	84

### 16 Months Follow Up of Patients’ Behavior Toward COVID-19 Infection and Risk Prevention Measures

**Figure 2:**
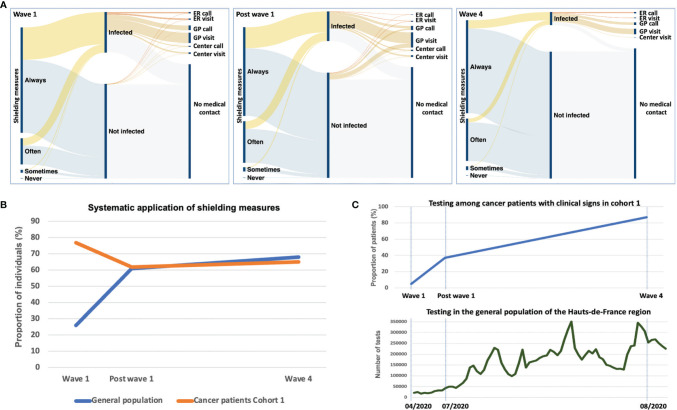
Patients’ behavior toward COVID-19 infection. **(A)** Sankey diagrams of patients’ application of shielding measures during the pandemic (cohort 1), and of the medical care sought by infected patients (experiencing COVID-19-like symptoms) and asymptomatic patients. **(B)** Systematic application of shielding measures in the general population of the Hauts-de-France region (blue line) and among cancer patients (cohort 1, orange line) during the pandemic. The systematic wear of the mask was the indicator used for the general population using the French GEODE database. Among cancer patients, the systematic application of five shielding measures globally (wearing a mask, avoiding gatherings, hand washing, physical distance, coughing in the elbow) was indicated. **(C)** Testing among cancer patients in cohort 1 (upper graph) and the general population of the French Hauts-de-France region (lower graph). The proportion of cancer patients with clinical symptoms of COVID-19 infection tested for COVID is indicated per wave. The trend of testing in the general population of the same region is indicated as the number of weekly tests during the pandemic. ER, emergency room; GP, general practitioner; Center, Oscar Lambret cancer center.

In cohort 1, 30% of the patients had COVID-like symptoms during the first wave while less than 10% were infected during the fourth wave (**Table 1** and **Figure 2A**). No association was observed at wave 1 between COVID-19 infection and the type of cancer care received (**Supplementary material**). Systematic application of shielding measures fell from 77% at wave 1 to 62% post wave 1 and 65% at wave 4, while the proportion of patients declaring they often apply them raised from 19% at wave 1 to 30% at wave 4. These measures were applied by most infected patients (**Figure 2A**). Patients promptly complied with the systematic application of shielding measures at the beginning of the pandemic (77%) compared to only 26% in the general population at wave 1 (**Figure 2B**). Afterward, the proportions were similar between cancer patients and the general population.

The Sankey diagrams in **Figure 2A** also showed that 49% of the patients with COVID-like symptoms did not seek medical advice during wave 1. This proportion was 42% after wave 1 and 25% during wave 4. When medical advice was sought, visits or calls to their general practitioner were the preferred options. The majority had mild disease with only a few visits to the emergency room or hospitalization.

Testing was made available in France in May 2020. In July 2020 (post wave 1), only 37% of the patients experiencing COVID-19-like symptoms were tested for confirmation. One year later, 87% of the patients with symptoms had a test to confirm the infection (**Table 1**). Testing for COVID-19 in cancer patients raised during the pandemic with a similar trend compared to the general population (**Figure 2C**).

**Figure 3:**
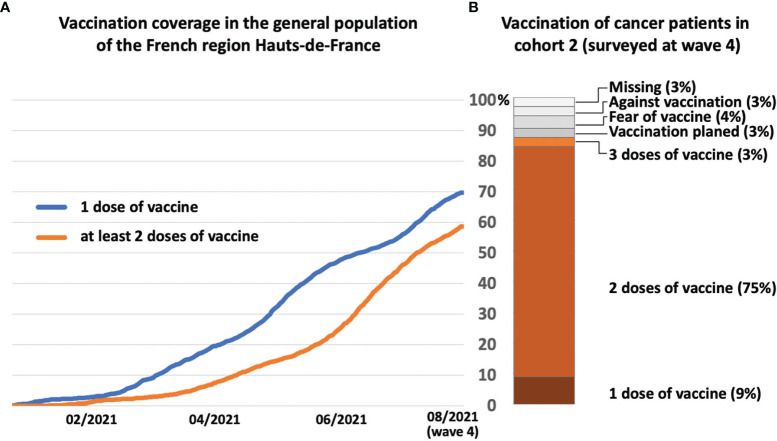
Vaccination **(A)** kinetics in the general population in the Hauts-de-France region and **(B)** in cancer patients (cohort 2) at wave 4 (august 2021), with the number of doses received. Lack of vaccination was distributed according to patients’ motivations.

Vaccination in France started slowly in January 2021 to reach 70% of the general population in Hauts-de-France receiving one jab and 60% two jabs by August 2021 (**Figure 3A**). In contrast, cancer patients in cohort 2 were committed to vaccination against COVID-19 with 87% of the patients receiving at least one dose of the vaccine and 84% receiving two doses (**Figure 3B**). However, 7% of cancer patients did not get vaccinated either because they feared the vaccines’ side effects or because they opposed vaccination (**Figure 3B**). The recent cancer care received had no impact on patients’ vaccination (**Supplementary material**).

### Short- and Longer-Term Clinical Patterns of Mild COVID-19 Infection

**Figure 4:**
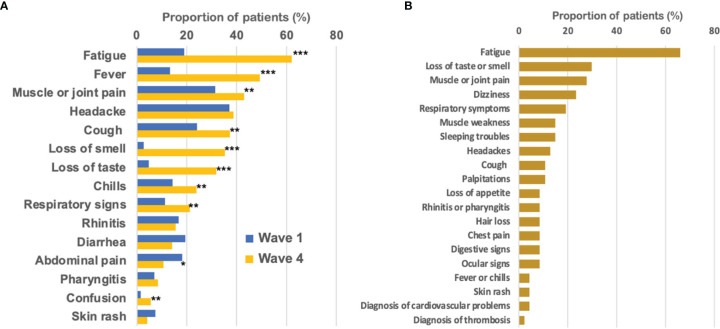
Clinical patterns of mild COVID-19 infection. **(A)** Symptoms associated with COVID-19 experienced by cancer patients in cohort 1 during wave 1 (in blue) and wave 4 (in orange). **(B)** Long-lasting COVID-19 symptoms among cohort 2 patients (at wave 4). *p<0.05; **p<0.01; ***p<0.001.

The most prevalent symptoms during wave 1 were muscle or joint pain (31%), headaches (37%), cough (24%), fatigue (19%), diarrhea (19%), and abdominal pain (18%) (**Figure 4A**). Muscle or joint pain was significantly more frequent in patients receiving endocrine therapy and targeted therapy (p=0.02). There was a non-significant trend for a higher frequency of chills in patients receiving immunotherapy (p=0.08) and a trend for a higher frequency of headaches in patients in follow-up or treated with endocrine therapy or who had surgery (p=0.06) (**Supplementary Material**).

The frequency of symptoms significantly differed during wave 4 compared to wave 1. A higher frequency of fatigue, fever, loss of smell, loss of taste, and respiratory signs was observed during wave 4 (**Figure 4A**). Most patients who tested positive for COVID-19 in cohort 2 (n=168) had mild disease; less than 10% reported hospitalization. Among infected patients, 22% (n=37) experienced at least one symptom lasting more than 6 months. Compared to other patients who tested positive, patients developing long-lasting symptoms were more frequently women (non-significant trend) and had more frequently a BMI over 30 or comorbidity, such as hypertension, cardiovascular disease, or asthma. Their cancer type, recent care, application of shielding measures, and vaccination status did not differ. They requested more often psychological support (**Table 2**).

**Table 2:** Patients’ characteristics among infected patients in cohort 2 according to long-lasting symptoms.

	Long-lasting signs		No long-lasting signs		
**Participants, n**	**37**	**%**	**131**	**%**	**p**
**Age, years**					0.46
Less than 50	9	24	33	25	
50-59	11	30	40	31	
60-69	13	35	32	24	
70+	4	11	26	20	
**Gender**					0.14
Female	30	81	90	69	
Male	7	19	41	31	
**BMI (kg/m2)**					0.02
<30	22	60	107	82	
30+	12	32	19	14	
Missing	3	8	5	4	
**Cigarette smoking**					0.86
Current smoker	3	8	7	5	
Former smoker	9	24	30	23	
Never-smoker	25	68	93	71	
Missing			1	1	
**Comorbid condition**					0.001
Yes	25	68	47	36	
No	9	24	47	36	
Missing	3	8	37	28	
**Comorbidities**					0.05
Hypertension	12	32	26	20	
Cardiovascular diseases	9	24	12	9	
Asthma	5	14	1	1	
Cancer type					0.58
Breast	18	49	61	47	
Gynecologic	5	14	10	8	
Digestive	2	5	6	5	
Head & neck	3	8	5	4	
Lung	1	3	9	7	
Urologic	2	5	10	8	
Sarcoma	0	0	8	6	
Missing	6	16	22	17	
**Recent care**					0.84
Follow up	17	46	73	56	
Chemotherapy	3	8	22	17	
Radiation therapy	4	11	13	10	
Surgery	1	3	14	11	
Endocrine therapy	4	11	14	11	
Targeted therapy	1	3	7	5	
Immunotherapy	1	3	5	4	
**Shielding measures**					0.43
Systematic	25	68	77	59	
Often	10	27	49	37	
Sometimes	2	5	3	2	
Never	0	0	0	0	
Missing			2	2	
**Vaccination**					0.32
No	11	30	35	27	
One dose	19	51	59	45	
Two doses	7	19	37	28	
**Hospitalization**	3	8	12	9	0.84
**Request for psychological support**	9	24	15	11	0.04
**Financial difficulty**	4	11	7	5	0.23

The most frequent symptoms associated with long-lasting COVID in cohort 2 were fatigue (66% of the patients), loss of taste or smell (30%), muscle or joint pain (28%), dizziness (23%), and respiratory signs (19%). Other symptoms experienced by more than 10% of the patients were muscle weakness, sleeping trouble, headaches, cough, and palpitations (**Figure 4B**).

### COVID-19 Impact on Patients’ Feelings and Behavior

**Figure 5:**
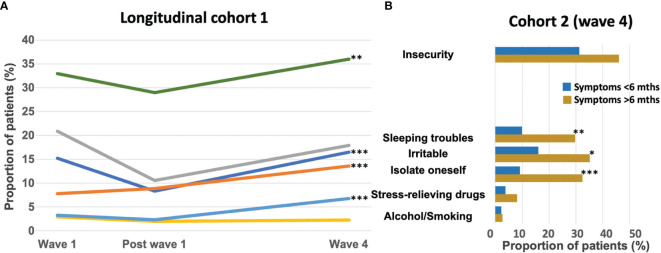
Proportion of patients in **(A)** cohort 1 (longitudinal cohort) experiencing insecurity, sleeping troubles, feeling irritable or the need to self-isolate, to consume stress-relieving drugs, alcohol, or tobacco during the pandemic at wave 1, post wave 1, and at wave 4. **(B)** Proportion of patients experiencing these feelings in cohort 2 (at wave 4) among patients tested positive according to the duration of COVID symptoms (less than 6 months in blue, or more than 6 months in brown). Mths: months; *p<0.05; **p<0.01; ***p<0.001.

More than 20% of the patients experienced sleeping troubles or nightmares during the first COVID wave. This proportion dropped to 10% after the first wave but then increased to reach 18% during the fourth wave. The same pattern was observed with feeling irritable. The isolation behavior almost doubled from wave 1 to reach 13% of the patients at wave 4. The consumption of stress-relieving drugs increased slightly during the pandemic from 3% to 7% of the patients at wave 4. Alcohol consumption and smoking remained stable (2% to 3%) during the pandemic. One-third of the patients reported feeling insecure throughout the pandemic with an increase at wave 4 (**Figure 5A**).

Patients experiencing long-lasting COVID-19 symptoms had a significantly higher frequency of sleeping troubles (30%), irritability (35%), or self-isolation (32%). They also had a trend toward a greater feeling of insecurity (46%) (**Figure 5B**).

## Discussion

Follow-up of cancer patients for as long as 16 months during the pandemic to inform patients’ behavior and the infection patterns in a population with predominantly mild disease are unique aspects of our cohort. Despite a high and early adherence to shielding measures, 30% of the patients were infected during the first wave. Vaccination was taken up by most patients, which accounts for the low prevalence of COVID-19 infection one year later. The changes in the clinical pattern observed between the first and the fourth waves may be explained by the different types of COVID-19 variants in circulation at these time points (9). The Delta variant was detected in the French population in May 2021. The higher frequency of fever, cough, loss of smell, muscle pain, and headaches observed in our cancer cohort during the fourth wave were also signs frequently reported in a group of healthy healthcare workers during an outbreak of Delta variant (10).

The persistence of signs developed during a COVID-19 infection is recognized as long COVID or post-COVID syndrome, in the absence of an alternative diagnosis. The current cut-off duration was set at 12 weeks (11). Little is known regarding the longer-term burden among cancer patients after a mild COVID-19 infection. Our study showed that a large proportion (22%) of cancer patients experienced persistent symptoms for more than 6 months after mild disease. This frequency is in the range of previous reports in non-cancer populations where 10% to 35% of the individuals had persistent signs for up to 3 months after mild COVID (12). In the spectrum of long-lasting signs in our cancer cohort, fatigue was the dominant symptom along with neurosensory dysfunction, muscle or joint pain, or respiratory signs, particularly in females and patients with higher BMI. Similar signs have been reported in non-cancer patients after mild to moderate acute COVID-19 infection (13, 14). A higher prevalence of long-lasting symptoms was also shown in females with increasing BMI (13, 15). The presence of more than five different symptoms during the first week of acute COVID-19 was a predictor of long covid risk in non-cancer patients (15). Models integrating clinical and biological markers have also been proposed (16, 17). Long COVID risk models specific to cancer populations still need to be addressed. The development of risk prediction models could help identify patients at higher risk of long COVID to implement novel treatment or prevention strategies.

These persistent symptoms had a negative impact on patients’ well-being, on top of the pandemic impact. Patients with persistent symptoms experienced significantly more sleeping troubles, self-isolation, increased consumption of stress-relieving drugs, or the need for psychological support. Loneliness was suggested to negatively affect patients’ mental health, especially in older patients (18). A systematic follow-up of infected cancer patients will help refine the clinical spectrum of long COVID and quantify the burden of additional physical and psychological morbidities and social consequences in this population. Information and training of healthcare workers are necessary to properly recognize long COVID and work with multidisciplinary teams to provide appropriate medical and rehabilitation support (5).

Our study showed disturbing results about patients’ behavior with regard to COVID-19 infection and risk prevention. Almost half of the patients with COVID-like signs did not seek medical advice during or after the first wave, which may have delayed cancer care. Moreover, 15 months later, still, 25% of the patients with symptoms had no medical contact. Although vaccination uptake in our study was higher than in the general population, approximately 10% of cancer patients in our cohort did not start vaccination. A French cohort study on the general public reported that 10% to 48% of people were not willing to be vaccinated, mostly among female, younger, lower-income or lower-education level individuals or ethnic minorities (19). The most common reasons for opposing COVID-19 vaccination in the general population were personal reasons, doubts about the vaccine efficacy, a lack of trust, or being anti-vax (20). Communicating about the expected benefits of vaccination was shown to be a key influential factor in decision making (21) and will be important to preserve patients’ adherence to repetitive injection schema and to convince patients refractory to vaccination.

Nevertheless, our results showed that vaccination was not associated with less long-lasting COVID symptoms, suggesting that the current vaccines may not confer protection against COVID-19’s longer-term burden in cancer patients. Although vaccination with three doses of mRNA vaccine has been associated with protection against both the Omicron and Delta variants (22), the high rates of long COVID in the literature question whether it confers protection or reduces long COVID. Moreover, a decline over 6 months in neutralizing antibody responses against the original SARS-CoV-2 and the Beta and Delta variants was shown in cancer patients and healthy individuals after two doses of mRNA vaccines (23, 24), justifying the third dose and questioning the fourth dose. The topic of vaccination impact on long COVID in cancer patients requires further investigation.

In this vulnerable population, the application of prevention measures against COVID-19 infection remains a safe policy. Our results showed that patients’ adherence to shielding measures declined over time, quickly after the first wave, even before the French government eased the wearing of masks outdoors. After the first wave, their feeling of insecurity was lower, and their perceived health risk may have followed a similar pattern. Risk perception was reported to influence adherence to shielding measures (25). Alerting care providers to maintain messages reinforcing prevention and vaccination toward cancer patients is important given the risk of misinformation in this vulnerable population. A study recently reported that cancer patients undergoing cancer treatment were more likely to believe false information about COVID-19 prevention and treatment compared to survivors not in active treatment or individuals with no cancer history (26).

Study limitations include a single study location, potential bias from self-reported symptoms, and disproportionate female contributors due to a large proportion of breast and gynecologic cancers. Long COVID embraces a wide spectrum of symptoms and organ involvement, with potentially challenging differential diagnoses in cancer patients such as treatment-related symptoms, disease symptoms, or depressive signs. However, in the present study, half of the long-lasting cases were patients in follow-up, which suggests that the symptoms were likely related to COVID-19.

In conclusion, our research showed that despite the presumable harmless clinical presentation of mild COVID-19, its consequences may extend far beyond the acute infection phase. In the vulnerable cancer population, it is critical to quantify the long-term burden of COVID-19 to provide adequate supportive care and allocation of resources. This work also highlights the need to pursue patients’ education and healthcare workers’ awareness of COVID-19 risks.

## Data Availability Statement

The raw data supporting the conclusions of this article will be made available by the authors, without undue reservation.

## Ethics Statement

The online survey was completely anonymous and in compliance with the European General Regulation (EU) No. 2016/679 on Data Protection (GDPR) of April 26, 2016. An information letter inserted before the questionnaires notified the participants that completing the questionnaire infers an agreement to their participation in the study and subsequently a written consent was not required. The study was examined and approved by the internal review board (IRB) at Oscar Lambret Cancer Center, Lille, France on April 16, 2020.

## Author Contributions

NH wrote the manuscript original draft. NH and SG designed the study. KL and SG collected the survey data. NH, KL, and SG performed the analyses. SL, SB, CB, EK, AB, and EL corrected the manuscript. NH, SG, and EL supervised the project and EL provided the funding. All authors contributed to the article and approved the submitted version.

## Funding

Funding was provided by Oscar Lambret Cancer center (Lille, France).

## Conflict of Interest

The authors declare that the research was conducted in the absence of any commercial or financial relationships that could be construed as a potential conflict of interest.

## Publisher’s Note

All claims expressed in this article are solely those of the authors and do not necessarily represent those of their affiliated organizations, or those of the publisher, the editors and the reviewers. Any product that may be evaluated in this article, or claim that may be made by its manufacturer, is not guaranteed or endorsed by the publisher.
