# Supplementary material for: 16 Months Follow Up of Patients’ Behavior and Mild COVID-19 Patterns in a Large Cohort of Cancer Patients During the Pandemic
Source: Front Oncol. 2022 Jun 7;12:901426. doi: 10.3389/fonc.2022.901426 (PMC9209649; doi:10.3389/fonc.2022.901426)

Supplementary Material

Supplementary Figure 1: Proportion of patients who experienced symptoms associated with covid-19 during wave 1 among cancer care groups.

30% of patients in follow up experienced covid-19 signs, 33% of patients in chemotherapy, 36% of patients in endocrine therapy, 27% of patients in targeted therapy, 37% of patients in immunotherapy, 29% of patients in surgery, and 26% of patients in radiation therapy. No significant association was observed between covid-19 infection and the type of recent care received by patients.

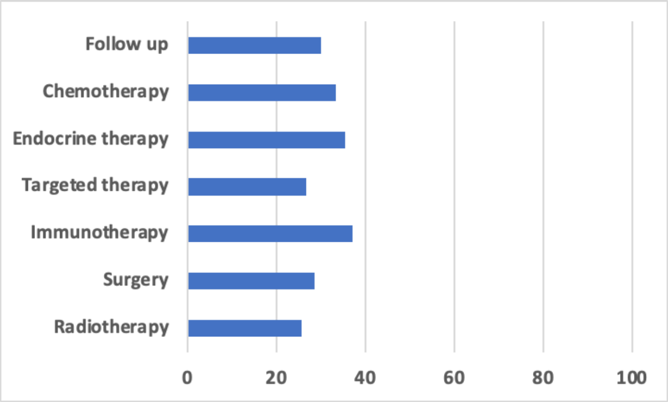

Supplementary Figure 2: Frequency of symptoms associated with covid-19 experienced by cancer patients in cohort 1 during wave 1 according to the recent care received: follow up, chemotherapy, endocrine therapy, targeted therapy, immunotherapy, surgery, or radiation therapy.

Muscle or joint pain was significantly more frequent in patients receiving endocrine therapy and targeted therapy (p=0.02). There was a non-significant trend for a higher frequency of chills in patients receiving immunotherapy (p=0.08) and a trend for a higher frequency of headaches in patients in follow-up or treated with endocrine therapy or who had surgery (p=0.06).

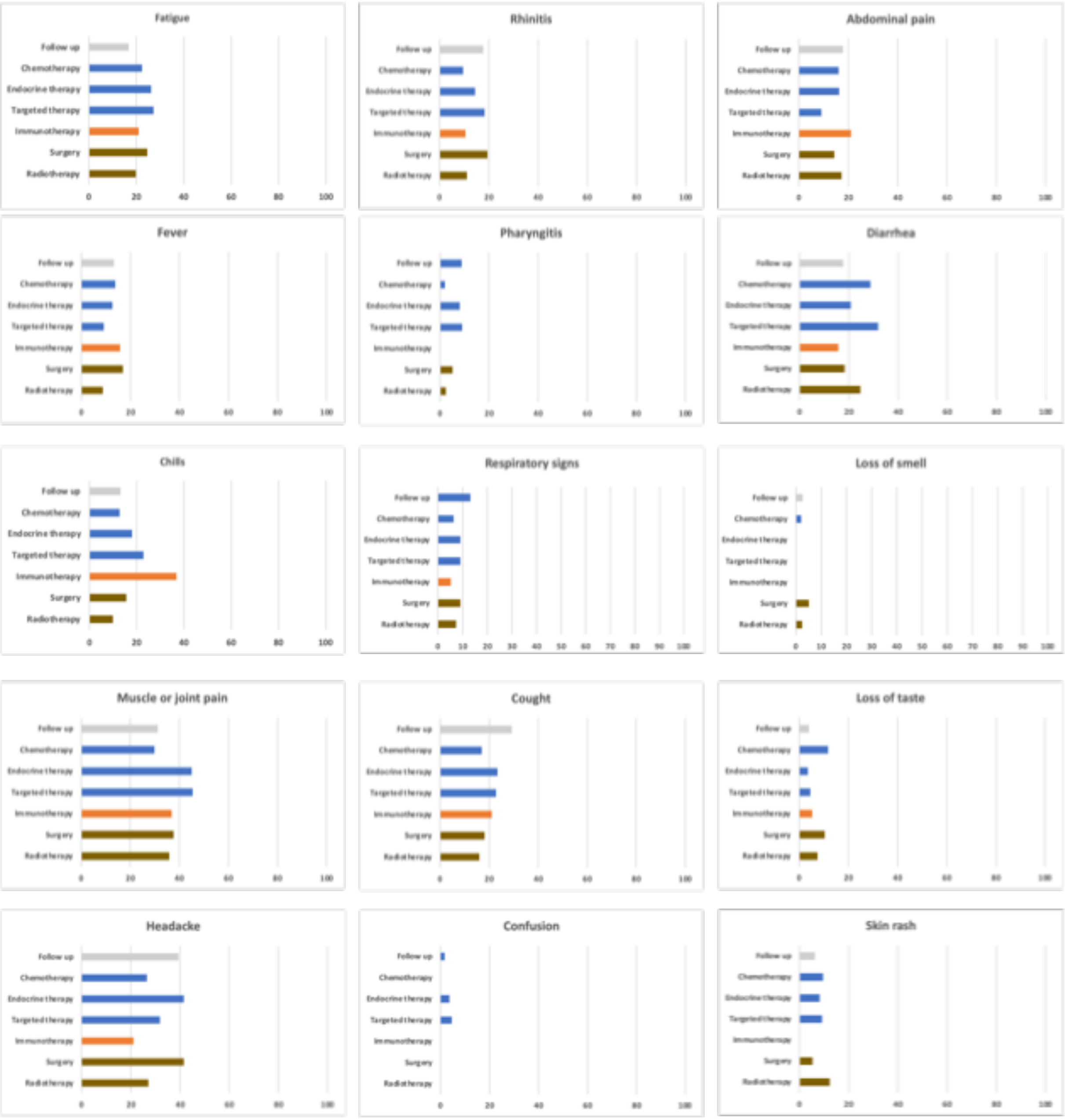

Supplementary Figure 3: Proportion of vaccinated patients according to the cancer care received.

More than 80% of the patients in cohort 2 were vaccinated independently of the care received.

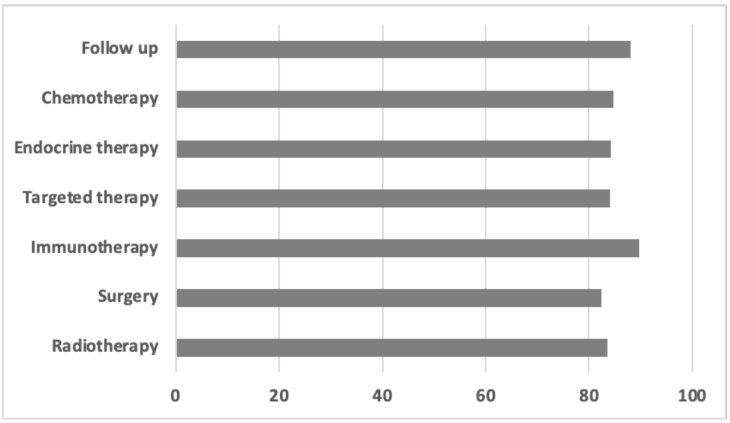

Supplement: Supplementary file 1 [file DataSheet_1.pdf]
